# Asymptomatic bacteriuria screening for developing countries using a modified water quality test kit

**DOI:** 10.1128/aem.01567-24

**Published:** 2024-10-30

**Authors:** Jolie A. Stocki, Rachel C. Fleck, Ivy B. Nguyen, Ryan Walde, Harry L. T. Mobley, Allyson E. Shea

**Affiliations:** 1 Tufts University School of Medicine, Boston, Massachusetts, USA; 2 Department of Microbiology and Immunology, University of South Alabama, Frederick P. Whiddon College of Medicine, Mobile, Alabama, USA; 3 Department of Pathology, University of South Alabama, Frederick P. Whiddon College of Medicine, Mobile, Alabama, USA; 4 Department of Microbiology and Immunology, University of Michigan Medical School, Ann Arbor, Michigan, USA; INRS Armand-Frappier Sante Biotechnologie Research Centre, Laval, Canada

**Keywords:** urinary tract infections, asymptomatic bacteriuria, pregnancy, *E. coli*

## Abstract

Between 2% and 15% of pregnant women unknowingly experience asymptomatic bacteriuria (ASB), defined as ≥10^5^ CFU per milliliter of urine in the absence of symptoms. ASB increases the risk of adverse pregnancy outcomes including pyelonephritis, preterm labor, and low-birth weight infants. While pregnant women in the United States are routinely screened for ASB, those in developing countries with limited resources and funding lack an accurate mechanism for ASB screening. Aquagenx water quality test kits detect *Escherichia coli*, the most common causative agent of ASB, and total coliform bacteria in drinking water via colorimetric and fluorescent indicators. We found that the Aquagenx system is compatible with human urine and then proceeded to develop an ASB screening protocol using disposable inoculating loops. Our protocol diagnosed artificial ASB^−^ samples (10^4^ CFU/mL *E. coli*) with a false positive (FP) rate of 33% (*n* = 18) and ASB^+^ (10^5^ CFU/mL *E. coli*) with a false negative (FN) rate of 5.6% (*n* = 18). Clinical sample testing with our protocol revealed a FP rate of 0% in ASB^−^ samples (*n* = 28) and a FN rate of 0% in ASB^+^ samples caused by coliforms (*n* = 13). Aquagenx did not detect ASB in nine clinical samples with non-coliform etiological agents due to the limitations of the technology. However, with very high accuracy for detection of *E. coli* and other causative agents that collectively account for 90.1% of ASB cases, these kits could be used as a diagnostic ASB screening tool in developing countries in which there is currently no alternative to urine culture.

**IMPORTANCE:**

Asymptomatic bacteriuria (ASB) affects 2%–15% of pregnant women and can result in adverse maternal and fetal outcomes if left undetected and untreated. In the United States and other developed nations, pregnant women are regularly screened for ASB via urine culture. However, in low-resource countries where bacterial culture is not available, dipstick testing is used. Although accurate in cases of symptomatic bacteriuria, dipstick detection is ineffective for detecting ASB. Here, we made use of an existing water quality field test for ASB urine screening, which would be readily deployable in low-resource settings. We optimized a dilution protocol for sampling patient urine within the detection limits of the Aquagenx kit technology. Overall, we were able to detect ASB samples with Gram-negative pathogens that collectively account for 90% of all ASB cases. Utilization of this repurposed technology for proactive medical screening may help prevent adverse pregnancy and birth outcomes due to ASB.

## INTRODUCTION

Asymptomatic bacteriuria (ASB) is defined as the presence of ≥10^5^ CFU/mL bacteria in urine without clinical symptoms and is most commonly caused by *Escherichia coli* (*E. coli*) (1, 2). No effective modalities currently exist to accurately diagnose ASB without urine culture; therefore, detection is extremely difficult in developing countries where the equipment required for bacterial culture is not readily available. In cases of symptomatic bacteriuria [urinary tract infection (UTI)], urine dipsticks are accurate in diagnosing infection and sufficient to merit the administration of treatment (3, 4). However, urine dipstick testing alone is not sufficient to establish bacteriuria in asymptomatic cases (ASB) because the sensitivity is only 33%–65% (3, 5). For example, one study found a FN rate of 53% when screening an obstetric population for ASB using dipsticks (6).

While ASB does not typically carry potential for harm, pregnancy is one of the few instances in which it can present with high risk (6). ASB occurs in an estimated 2%–15% of pregnant women globally (7) and is clinically associated with preterm birth and low infant birth weight (1, 7). When untreated, ASB progresses to UTI or acute pyelonephritis in nearly one-third of pregnant women, which can result in increased fetal and maternal morbidity and mortality (1, 7, 8). Acute pyelonephritis in pregnancy can result in sepsis, acute respiratory distress syndrome, and anemia. In severe, progressive cases, it has been shown to cause preterm delivery in over 20% of cases and miscarriage in 10% of cases (1, 7–9). Approximately 51%–70% of cases of acute pyelonephritis due to ASB in pregnancy is caused by *E. coli*, 13% by other coliforms, and 10%–12% by Gram-positive organisms (2, 8).

Due to these prominent risks, the Infectious Diseases Society of America strongly recommends proactively screening for and treating ASB in pregnant women (1). Screening typically occurs at least once in the first trimester and does not need to be repeated as there is only a 1%–2% risk of later developing ASB after a negative initial result (10, 11). The diagnosis is made based on two consecutive voided urine specimens with ≥10^5^ CFU/mL of a single uropathogen in an asymptomatic patient (1). Once ASB has been diagnosed, it is typically treated similarly to cystitis with a 4–7-day course of antibiotics (1). Screening, detection, and subsequent treatment of ASB early in pregnancy have been found to reduce the incidence of pyelonephritis from 20%–35% to 1%–4% (7).

Here, we demonstrate an accurate alternative ASB screening mechanism using Aquagenx water quality test kits. Aquagenx is commonly used in low-resource settings to test drinking water for the presence of *E. coli* and other coliform bacteria. The protocol involves adding 100 mL of the test water sample to an Aquagenx bag, adding Aquagenx proprietary growth media, and incubating for the amount of time specified in the guidelines depending on the ambient temperature. These kits accurately identify the presence of *E. coli* in water samples and have received high marks for reliability and usability for drinking water testing by the World Health Organization (WHO) (12). Aquagenx test kits are an ideal candidate for detecting *E. coli* in human urine samples in low-resource settings lacking access to urine culture because they are simple to use, can be incubated outdoors, require few resources, and can give results within a day.

This study demonstrates a highly accurate, low-resource feasible ASB screening method using Aquagenx water quality test kits. Screening mock urine samples (*n* = 36) for ASB using Aquagenx resulted in a FP rate of 33.3% and a FN rate of 5.56%. The FP rate was determined with ASB^−^ samples prepared at 10^4^ CFU/mL, just under the clinical threshold for ASB. Screening hospital-acquired urine samples from pregnant women (*n* = 40) with Aquagenx detected all ASB^+^ cases caused by coliform bacteria. Clean-catch urine from uninfected individuals (*n* = 5), often containing native perineal flora, did not result in any FPs. In addition to the identification of the major etiological agent of ASB via a colorimetric indicator, the kit is also effective at detecting other Gram-negative coliforms under UV light. Collectively, this method can detect 90% of ASB causative species (13). Our work has the capability to provide remote and low-resource settings with access to ASB screening technologies and diagnostics that far surpass their current methodologies.

## RESULTS

### Validation of Aquagenx technology with human urine

**Fig 1:**
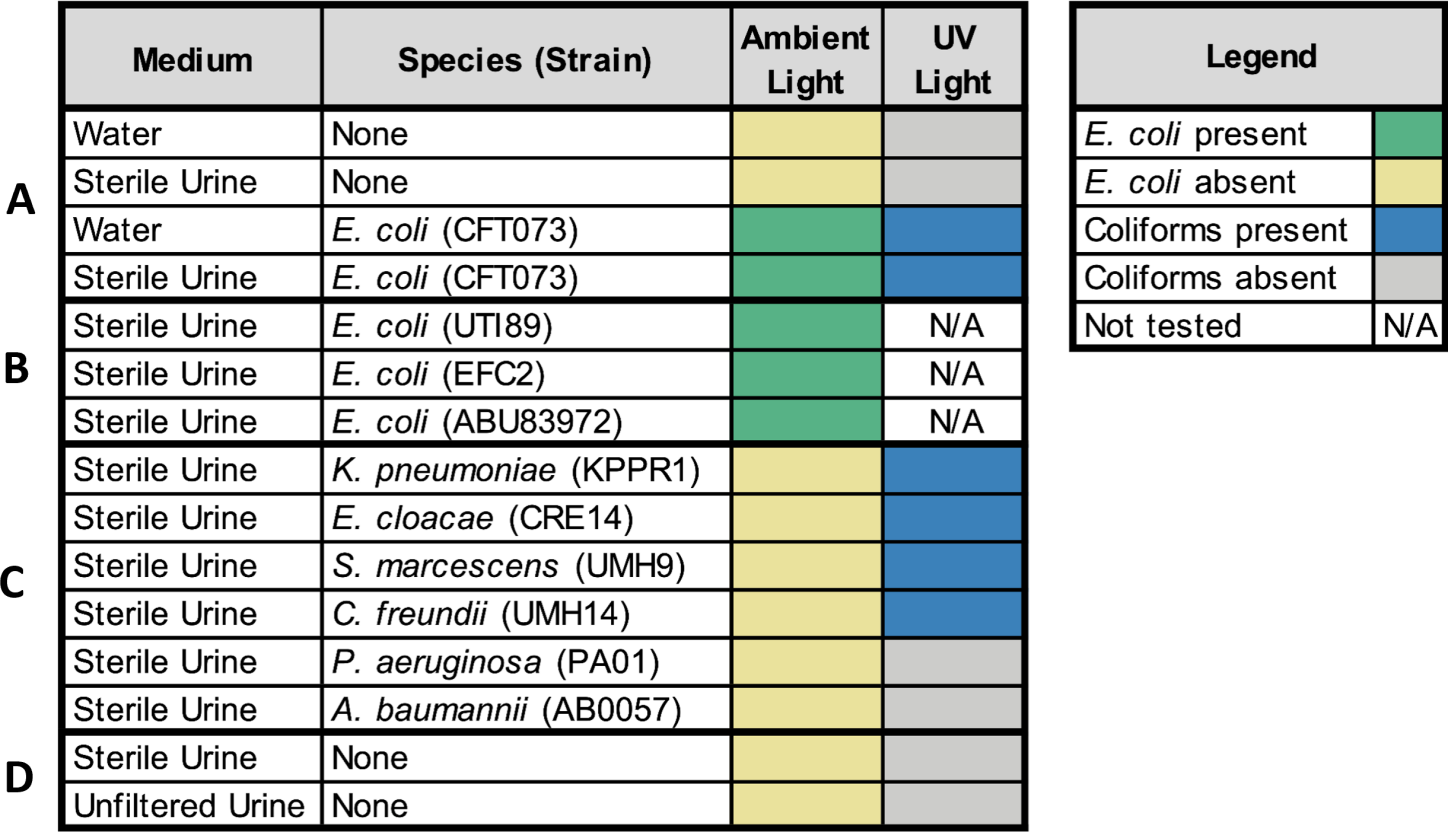
Validation of Aquagenx technology with human urine. A series of validation experiments were performed with Aquagenx kits using human urine as a base medium. (**A**) Filter-sterilized pooled human urine was tested alone in the bag with and without *E. coli* CFT073. Sterile water alone served as a negative control (yellow), and sterile water with *E. coli* CFT073 was a positive control (green). (**B**) Three additional *E. coli* pathotypes were tested in the Aquagenx kit and all yielded positive (green) results. (**C**) Other Gram-negative bacterial species commonly associated with ASB were also tested. As expected, all were negative for the presence of *E. coli* (yellow). Coliform species fluoresced under UV light (blue). (**D**) Unfiltered clean-catch urine provided from an uninfected individual was negative for color change and UV.

The Aquagenx water quality test kit is designed to indicate as little as 1 CFU per 100 mL of drinking water (0.01 CFU/mL) via colorimetric and fluorescent indicators (Fig. S1). We sought to leverage this simple technology for the diagnostics of UTI, specifically in the context of ASB. First, we had to validate the compatibility of the technology with human urine as the medium in lieu of water. We found that filter-sterilized human urine did not trigger the colorimetric or fluorescent indicator (Fig. 1A). Since *E. coli* is extremely genetically heterogeneous (14, 15), we decided to confirm colorimetric compatibility with *E. coli* strains of diverse origin. Uropathogenic *E. coli* (CFT073), intestinal *E. coli* (EFC2), and asymptomatic *E. coli* (ABU83972) strains all triggered colorimetric change in the Aquagenx bag (Fig. 1B). In fact, we were able to confirm that this colorimetric change is very specific to *E. coli*, and a variety of other bacterial coliform species known to cause ASB can also be detected via fluorescence under UV light (Fig. 1C). Aquagenx offers affordable UV flashlights as an optional add-on with purchase for simple UV detection in low-resource settings (16). Finally, unfiltered clean-catch human urine did not generate a color or fluorescent change (Fig. 1D). Collectively, these validation studies justify the use and utility of the Aquagenx technology for screening human urine. Aquagenx offers two test kits for detection of *E. coli* and total coliform bacteria: a Presence or Absence (P/A) kit and a Most Probable Number (MPN) kit. We first began developing an ASB screening protocol with the MPN kit.

### Testing an ASB screening protocol with MPN kits

**Fig 2:**
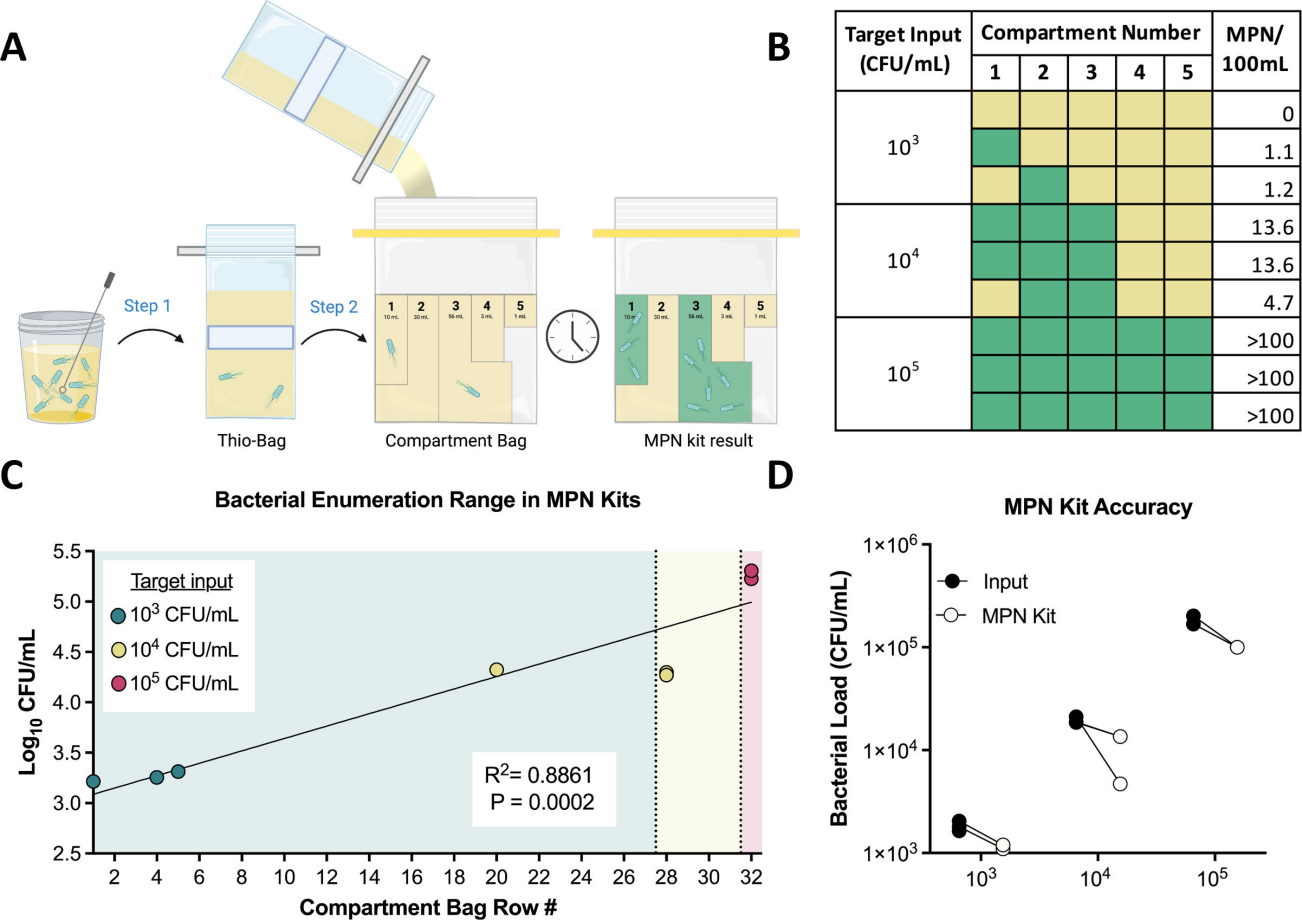
Validating the MPN kit ASB screening protocol with clinically relevant mock samples. (**A**) Graphic depicting the MPN kit ASB screening protocol. (**B**) Mock *E. coli* urine samples were prepared at 10^3^, 10^4^, and 10^5^ CFU/mL (*n* = 3) and subjected to our MPN kit ASB screening protocol. The bag compartments were scored in ambient light, where a positive result was a color change from yellow to green. The MPN of *E. coli* in 100 mL was determined using the Aquagenx MPN kit color chart (17). (**C**) Linear regression of the Log_10_ CFU of the input samples compared with MPN chart row number with displayed *R*^2^ and *P* value. Based on the MPN color chart and our dilution protocol, expected row number ranges for 10^3^ (shaded blue), 10^4^ (shaded yellow), and 10^5^ (shaded pink) CFU/mL bacterial loads were determined. (**D**) Actual input concentrations (black circles) are directly compared with output bacterial load (white circles) as determined by MPN kit results (Fig. S4).

Aquagenx MPN kits enumerate the bacteria in a 100-mL sample via a multi-compartmented bag with a detectable range of 1–100 CFU (0.01–1 CFU/mL) (17) (Fig. 2A). To limit the number of sample manipulations, we designed a protocol using a single dilution step. In this protocol, a sterile plastic inoculating loop is transferred into a Thio-Bag (Step 1); then, the contents of the Thio-Bag are poured into a Compartment Bag (Step 2) (Fig. 2A). This protocol was designed to achieve a color change in all five compartments of the bag for ASB^+^ (10^5^ CFU/mL) samples. Our calculations indicated that a 1-µL inoculating loop was needed to transfer 100 CFU from 10^5^ CFU/mL (ASB^+^) samples and 1 CFU from 10^3^ CFU/mL (ASB^−^) samples. To test this, we made mock urine with a concentration of 10^3^ CFU/mL and tested multiple inoculating loop volumes, 1 µL, 1 µL twice, and 10 µL, in Step 1 (Fig. S2). We simulated Thio-Bag inoculation by transferring an inoculating loop from the urine sample onto an Luria broth (LB) agar plate, and a pipetted volume served as the control. We found that, as expected, a 1-µL volume was the most accurate at transferring a single CFU to an LB agar plate (Fig. S2). We then proceeded to test 10^3^, 10^4^, and 10^5^ CFU/mL in the MPN kit (Fig. S3). The protocol used for these experiments is depicted in Fig. 2A. Each bacterial concentration was tested multiple times, and the colorimetric indicator outcomes were used to determine a MPN value for each sample (Fig. S4). We found that only 2/3 of samples at the lower limit of 10^3^ CFU/mL were detected, but all samples at 10^4^ and 10^5^ CFU/mL were detected by the colorimetric technology (Fig. 2B). These assigned MPN row numbers were plotted against the exact input CFU value to determine the linearity between actual versus expected outcomes (Fig. 2C). There was a significant positive correlation (*R*^2^ = 0.8861) between the input sample concentration, and the output value was determined by Aquagenx. Finally, these outcome measures were converted from an MPN value to CFU burden (Fig. S4) to determine the accuracy of the kit (Fig. 2D). The MPN kit had a 56-60% recovery rate, independent of the starting input CFU burden. This suggests that the current protocol underestimates the bacterial burden, likely due to the transfer of the sample from the Thio-Bag into the Compartment Bag for testing. Aquagenx makes an additional kit, which allows for direct testing in the Thio-Bag, termed the P/A kit. Thus, we decided to alter our protocol for application with the P/A kit.

### Development of an optimized ASB screening protocol with P/A kits

**Fig 3:**
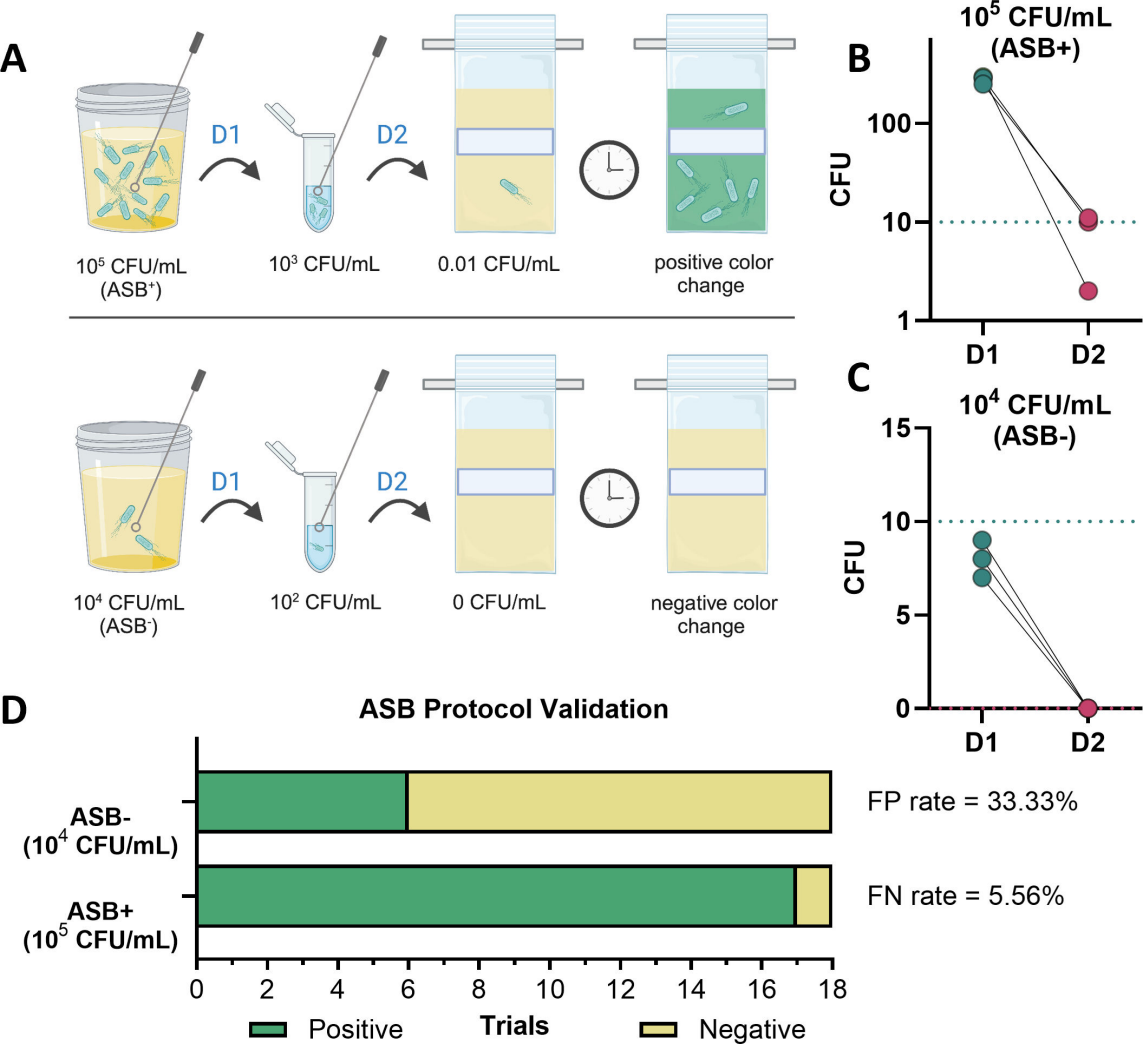
Validating the P/A kit ASB screening protocol with mock urine samples. (**A**) Depiction of the P/A kit ASB screening protocol. Artificial human urine samples with either (**B**) 10^5^ CFU/mL (ASB^+^) or (**C**) 10^4^ CFU/mL (ASB^−^) concentrations of uropathogenic *E. coli* CFT073 were subjected to our final optimized P/A kit protocol. After D1, CFU were determined by spread plating 100 µL from the microtube onto LB agar plates (green circles). D2 CFU output was determined by streaking 1 × 10 µL AT loop from the microtube onto an agar plate for enumeration (pink circles). Green dashed lines indicate the ideal CFU in D1, and pink dashed lines indicate the ideal CFU in D2. Experiments were repeated three times with three separate mock urine samples (*n* = 3). (**D**) Artificial urine samples with 10^5^ CFU/mL (ASB^+^) or 10^4^ CFU/mL (ASB^−^) were tested using Aquagenx P/A kits. Experiments were conducted in technical triplicate with six biological replicates (*n* = 18). The green color indicates a positive bag result, and the yellow color indicates a negative result.

Aquagenx is designed to identify as little as 0.01 CFU/mL, but the clinical definition of ASB is ≥10^5^ CFU/mL (1). Thus, a urine dilution protocol was required to transfer ≥1 CFU into the P/A kit from ASB^+^ samples while transferring 0 CFU from ASB^−^ samples (Fig. 3A). We proceeded to develop and optimize a urine dilution protocol suitable for low-resource settings using sterile disposable inoculating loops and microtubes. First, the transfer of a 10-µL inoculating loop from a 10^5^-CFU/mL urine specimen into a microtube of 990 µL sterile water (termed D1) was performed to dilute the sample 1:100 (Fig. 3A; Fig. S5). Different brands of loops were tested for optimization, with a target spread plate readout of >100 CFU corresponding to a concentration of 10^3^ CFU/mL in the microtube after D1 (Fig. S5). The Argos Technologies (AT) 10-µL loop used twice (total volume 20 µL) was the only condition tested that surpassed the optimal target concentration in the microtube (Fig. S5). We then proceeded to test the second dilution step (termed D2), where another sterile 10-µL inoculating loop was transferred from the microtube into the Aquagenx P/A bag (Fig. 3A). We generated a testing matrix, testing multiple D1 protocols (Fig. S5) with the same series of D2 dilutions (Fig. S6). Our target was to transfer 1–10 CFU into the bag to turn it blue/green at urine *E. coli* concentrations of ≥10^5^ CFU/mL but remain yellow at concentrations < 10^5^ CFU/mL (Fig. 3A). Ultimately, the 10-µL AT loop was used a single time from the microtube to the P/A bag (D2), as this protocol had the highest accuracy rate at 88.8% (Fig. S6). These data were used to determine our final P/A kit protocol (Fig. 3A): an Argos Technologies 10-µL loop used twice (total volume 20 µL) into the microtube, followed by another 10-µL AT loop used a single time from the microtube into the P/A bag.

### Assessing the screening protocol accuracy with mock ASB samples

To assess the accuracy of our optimized protocol (Fig. 3A), we tested both 10^5^ CFU/mL (ASB^+^) and 10^4^ CFU/mL (ASB^−^) artificially created urine samples with *E. coli*. We assessed the CFU burden after both D1 and D2 dilution steps to ensure that the final ASB^+^ sample would yield 1–10 CFU in the P/A bag, thus generating a positive result (Fig. 3B). This same protocol was repeated with an ASB^−^ sample that should yield no detectable CFU after D2, ensuring a negative result (Fig. 3C). After confirming via plate counts for total CFU, we moved to validate this protocol using the Aquagenx P/A bags. Samples were prepared on 6 different days, using technical triplicates for each dose (10^4^ and 10^5^ CFU/mL). In total, 36 samples were tested to determine the FP and FN rates (Fig. 3D). Our FP rate of ≥10^4^ CFU/mL (ASB^−^) turning the bag indicator was 33.3%, while our FN rate for missed ASB detection (≥10^5^ CFU/mL) was only 5.6%.

### Evaluating the clinical utility of urine screening with Aquagenx on patient samples

**Fig 4:**
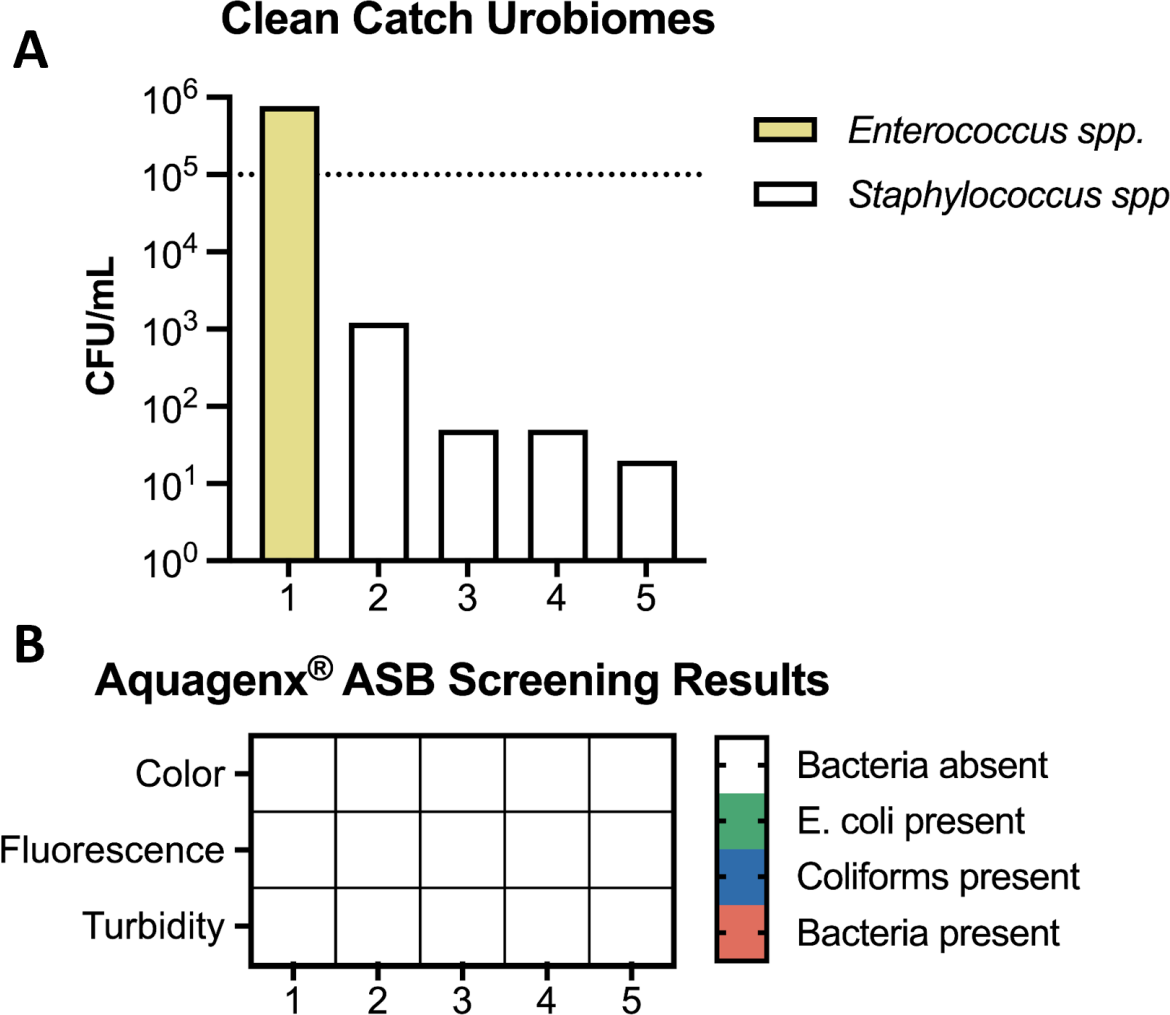
Testing Aquagenx P/A kit ASB screening protocol on clean-catch uninfected urine. (**A**) De-identified clean-catch urine samples (*n* = 5) from healthy women were acquired and drip plated on LB agar plates to enumerate the contaminating species present. Species were identified via matrix-assisted laser desorption ionization-time of flight (MALDI-TOF) mass spectrometry. (**B**) These samples were then subjected to our ASB screening protocol to determine whether normal flora alone could result in a FP result. Aquagenx bags were scored in ambient light where no color change (white) was a negative result for the presence of bacterial species, color change (green) was a positive result for the presence of *E. coli*, and turbidity was an indicator of non-coliform bacterial presence (orange). Bags were also inspected under UV light for fluorescence (blue) indicating the presence of coliform bacteria.

Up until this point, all samples had been artificially prepared by spiking known concentrations of bacteria into either sterile human urine or water. For full functionality, we needed to confirm that clean-catch human urine samples would not result in FPs. Five healthy, female, de-identified volunteers provided clean-catch urine samples that were subjected to our P/A kit protocol (Fig. 4). There was determined bacterial abundance in all samples, ranging from 10^1^ to 10^6^ CFU/mL. Bacterial composition consisted of *Staphylococcus* and *Enterococcus* species (Fig. 4A), which are estimated to cause 5.3% and 4.7% of ASB cases (13), respectively, but are also members of the commensal flora (18, 19). Although sample #1 had >10^5^ CFU/mL of a single bacterial species (*Enterococcus*) in the urine, Aquagenx technology did not detect these non-coliform bacteria (Fig. 4B). However, overall, the colorimetric and fluorescent results of the Aquagenx bags corresponded to the expected outcomes; none were positive because coliforms were not present at >10^5^ CFU/mL. We, therefore, demonstrated the accuracy of this protocol while accounting for any contamination by vaginal and dermatologic commensal organisms in self-collected urine samples.

**Fig 5:**
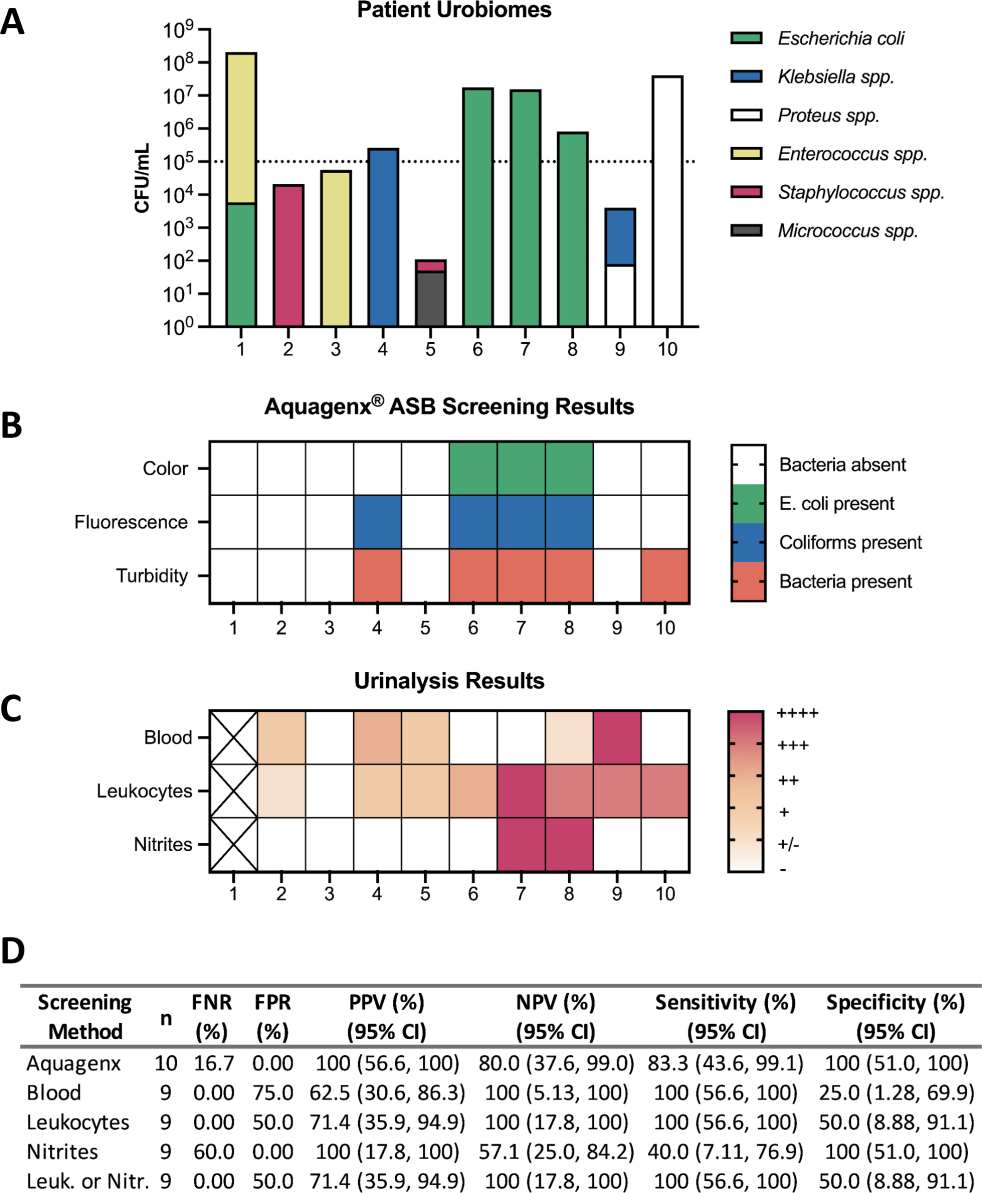
Testing Aquagenx P/A kit ASB screening protocol on clinical human urine specimens. De-identified clinical urine samples (*n* = 10) were obtained from the University of South Alabama Hospital. (**A**) Samples were plated on LB agar for enumeration and sent to MALDI-TOF for species identification to determine the urobiomes of each patient. (**B**) The samples were subjected to our established ASB screening protocol where Aquagenx bags were scored in ambient light and UV light for detection of ASB (10^5^ CFU/mL) concentrations of *E. coli* and total coliform bacteria. Turbidity was also assessed as an indicator of the overall presence of non-coliform bacteria in the sample. (**C**) Urine dipstick testing was performed at the University of South Alabama Hospital. Reported values include nitrites, leukocyte esterase, and detectable blood in the urine. The scale bar indicates the strength of the result, with dark-red being the highest value. White indicates a negative result; black X indicates missing values. (**D**) Multiple statistical metrics were computed for individual urine dipstick indicators and Aquagenx. Determined metrics have a reported 95% confidence interval (CI) with upper (UL) and lower (LL) limits.

We then acquired 10 de-identified patient urine samples from the University of South Alabama Hospital from uninfected (*n* = 5) and UTI^+^ (>10^3^ CFU/mL uropathogen) (*n* = 5) individuals (Fig. 5). Each sample was processed for urobiome identification and then subjected to our validated P/A kit protocol. Among the uninfected samples (#1–5), there was a variety of bacterial species, but none contained *E. coli* at a concentration of >10^5^ CFU/mL (Fig. 5A). Accordingly, none of the Aquagenx bags turned green/blue, but sample #4 was accurately UV positive due to >10^5^ CFU/mL of *Klebsiella* species (Fig. 5B ; Fig. S7). Sample #1 remained negative despite >10^5^ CFU/mL *Enterococcus* due to technology limitations of the kit. This was also observed in clean-catch sample #1 (Fig. 4A). Of the UTI^+^ samples (#6–10), three of five turned the bag color green/blue as expected due to confirmed >10^5^ CFU/mL abundance of *E. coli* (Fig. 5B; Fig. S7). Sample #9 had *Klebsiella* species < 10^5^ CFU/mL and thus did not emit fluorescence (Fig. 5B; Fig. S7). Sample #10 had *Proteus mirabilis* at >10^5^ CFU/mL but was not detected by Aquagenx colorimetric or UV technology. However, the bag did exhibit turbidity, which indicates a non-coliform Gram-negative bacterial presence above the ASB threshold.

We were able to obtain urine dipstick values for almost all patient samples (Fig. 5C). These data demonstrate the wide variability of clinical presentation. Dipstick results are not reliable for the screening of ASB, largely due to the lack of inflammation and corresponding symptoms (20). This is the primary purpose for our study, to provide a more reliable screening method that can be used in conjunction with, or in place of, dipstick tests in low-resource settings. Blood and leukocyte indicators detected ASB^+^ samples with 100% sensitivity but very low specificity (25% and 50%, respectively) (Fig. 5D). In contrast, nitrite indicators were much more sensitive (100%) but less specific (40%) (Fig. 5D). Sample #6 demonstrates a patient with >10^5^ CFU/mL *E. coli* in their urine and a positive leukocyte dipstick result; however, this dipstick result alone provides no bacterial species information to inform antibiotic administration. This is an example of a case in which the species-specific information provided by Aquagenx complements dipstick screening results.

### Aquagenx accurately detects ASB caused by coliform bacteria in pregnant patients

**Fig 6:**
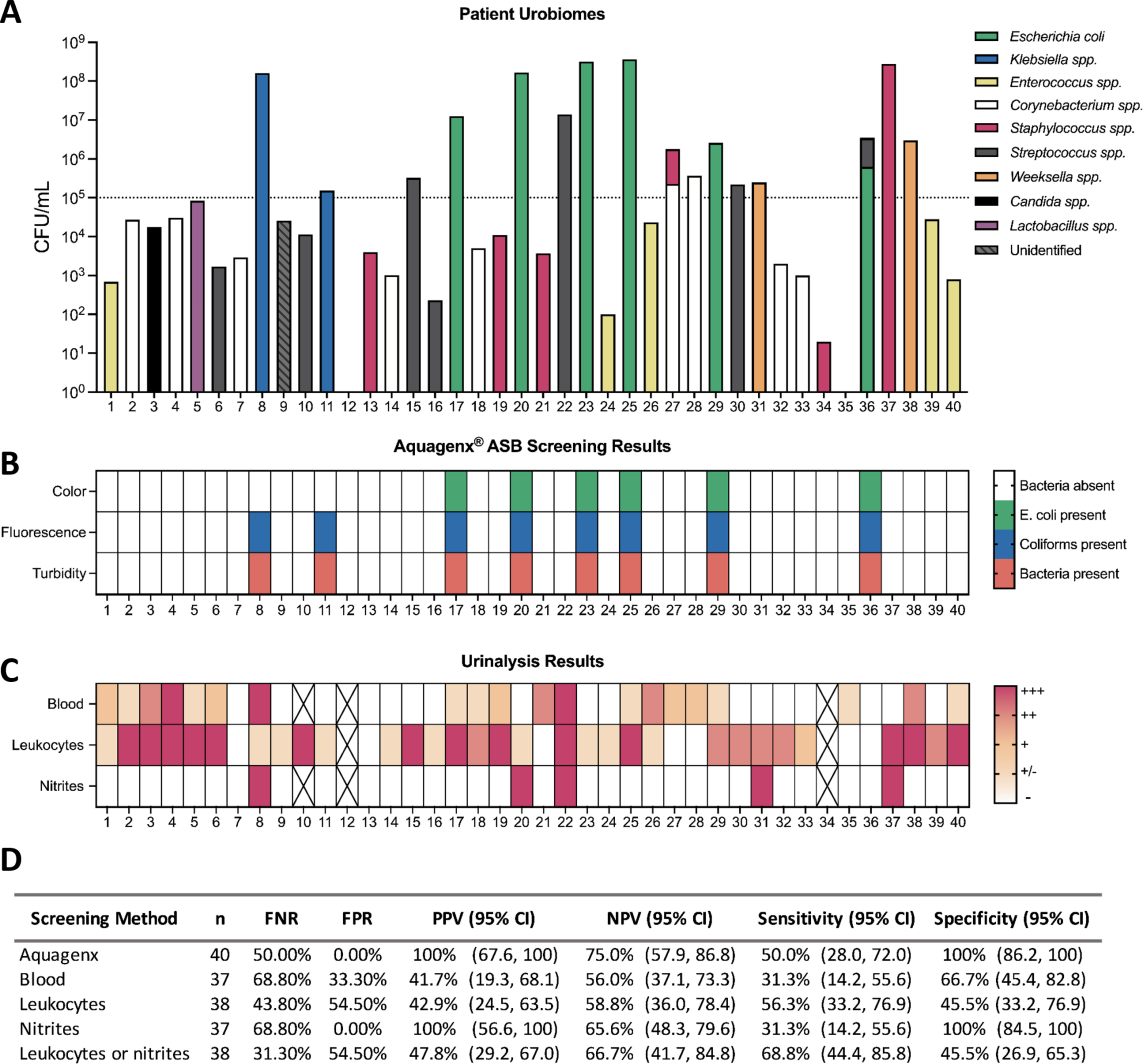
Testing Aquagenx P/A kit ASB screening protocol on human urine from pregnant patients. De-identified urine samples from pregnant patients (*n* = 40) were acquired from the University of South Alabama Hospital. (**A**) These samples were spread and drip plated on LB agar and blood agar to enumerate and isolate bacterial species; then, species identification was performed via MALDI-TOF. The bacterial species are represented by stacked bars with individual colors for each species. (**B**) ASB screening was performed on each sample using our established protocol where Aquagenx bags were scored by color change (green), fluorescence (blue), and turbidity (orange). (**C**) Urinalysis was performed at the University of South Alabama Hospital. Reported values include nitrites, leukocyte esterase, and detectable blood in the urine, and exact values are documented in Data Set S1. The scale bar indicates the strength of the result, with dark-red being the highest value. White indicates a negative result; black X indicates missing values. (**D**) Multiple statistical metrics were computed for individual urine dipstick indicators and Aquagenx. Determined metrics have a reported 95% CI with UL and LL.

Because detection of ASB is critical during pregnancy (6), we tested our P/A kit protocol with de-identified urine samples (*n* = 40) from independently confirmed pregnant patients at the University of South Alabama Hospital (Fig. 6). Of the 40 samples, 16 exhibited bacterial concentrations of ≥10^5^ CFU/mL (Fig. 6A). Samples #17, #20, #23, #25, #29, and #36 contained *E. coli* at ASB^+^ concentrations and accordingly both turned the bag color green and fluoresced under UV light (Fig. 6B). *Klebsiella* species were present at >10^5^ CFU/mL in samples #8 and #11, and these bags remained yellow, exhibited turbidity, and emitted fluorescence under UV light as expected. Samples #15, #22, #27, #28, #30, and #37 contained ASB^+^ concentrations of non-coliform, Gram-positive organisms; therefore, the bags exhibited no color change, fluorescence, or turbidity (Fig. S8). These results were expected considering the Aquagenx growth medium is coliform specific; however, the limitation of the Aquagenx technology did result in FN ASB screening results for these six samples. It is important to note that though our population sample of ASB^+^ samples contained 33.3% Gram-positive organisms, the literature cites that only about 10%–12% of all reported ASB cases is caused by these species (2, 9).

Dipstick indicators were less accurate than Aquagenx for ASB detection in obstetric patients (Fig. 6C). For example, blood and nitrite indicators missed detection of more ASB^+^ samples than Aquagenx and resulted in FN rates of 68.8% (Fig. 6D). Leukocyte esterase indicators detected ASB with slightly higher sensitivity (56.3% vs 50%) but significantly lower specificity (45.5% vs 100%) in comparison to Aquagenx (Fig. 6D). Detection of ASB by either a positive leukocyte esterase or nitrite indicator improved sensitivity (68.8%) compared with either indicator alone, but these metrics in combination still yielded a very low specificity (45%). These data together demonstrate the ability of Aquagenx to address some of the limitations with the current use of dipstick testing for ASB in low resource settings.

## DISCUSSION

This study demonstrates that Aquagenx water quality test kits, while most commonly used for the detection of fecal contamination in drinking water in low-resource settings (12), are compatible with human urine for accurately detecting *E. coli* via a colorimetric indicator and other uropathogenic coliforms via UV light (Fig. 1). We developed a two-step dilution protocol using 10-µL Argos Technologies loops dipped twice into the initial human urine sample and then once into the dilution tube to detect *E. coli* at concentrations > 10^5^ CFU/mL using Aquagenx P/A kits. We found an FP rate of 33.3% and an FN rate of 5.6% for the detection of *in vitro* clinical and subclinical ASB *E. coli* concentrations using this protocol (Fig. 3). Additionally, our protocol was able to accurately detect *E. coli* and other coliforms at clinical ASB concentrations in patient urine samples, remaining negative for all subclinical concentrations and non-coliform infections (Fig. 5 and 6). This protocol allows Aquagenx to become a feasible and accurate tool to screen for ASB during pregnancy in low-resource settings where urine culture is unavailable.

This P/A kit protocol has a very low FN rate (5.6%) for detection of *E. coli* at concentrations ≥ 10^5^ CFU/mL while utilizing a feasible method for low-resource settings at a low cost. Our protocol demonstrated 50% sensitivity and 100% specificity for ASB detection during clinical sample testing. When tested on ASB^−^ patient samples, the protocol resulted in a FP rate of 0% (*n* = 27). We attribute the elevated *in vitro* FP rate (33.3%) to testing only highly concentrated ASB^−^ samples (10^4^ CFU/mL), just under the clinical threshold for ASB (10^5^ CFU/mL) (1). In practice, a FP may result in giving unnecessary antibiotics to a pregnant patient (21), while a FN may result in low infant birth weight or pyelonephritis in pregnant patients (1, 7). Patients with 10^4^ CFU/mL, although subclinical, may be at risk of developing ASB. However, to our knowledge, no clinical studies have been conducted to show how frequently specific sub-threshold bacterial loads in urine progress to ASB, UTI, or pyelonephritis. Given the risks associated with ASB in pregnancy (1, 7), we feel it is most important to minimize the likelihood of type II error (FN) with this protocol and are therefore content with the 33.3% *in vitro* FP rate at 10^4^ CFU/mL of uropathogen. However, it is important to also note that unnecessary administration of antibiotics contributes to antimicrobial resistance. Clinical guidelines recommended screening two consecutive voided specimens for diagnosis (1); thus, a second screening test could decrease rates of FPs at sub-clinical concentrations and reduce the likelihood of unnecessary antibiotic administration.

Most non-*E*. *coli* Gram-negative organisms will grow and change the media from translucent to opaque (without a color change), which could indicate the need for further testing. This was demonstrated by UTI^+^ sample #10 (Fig. 5), which had a bacterial concentration of 10^7^ CFU/mL of *Proteus* spp. and generated turbidity in the Aquagenx bag. *Pseudomonas* spp. also generated turbidity when tested (Fig. S8). Samples #31 and #38 contained >10^5^ CFU/mL of *Weeksella* spp. (Fig. 6) and were the only Gram-negative ASB samples undetected by Aquagenx. However, *Weeksella* spp. are not a known common causative agent of ASB (8, 13) but have been isolated as a commensal from the genitourinary tract (22–24). Additionally, while the samples in our study had a high rate of Gram-positive species at ASB^+^ concentrations, these species have been shown to represent only a small fraction (10%–12%) of ASB-causing organisms (8). We hypothesize that this discrepancy is likely due to contamination during sample collection, which is complicated by the physiological changes experienced during pregnancy (25). A second screening test could eliminate some of these previously determined ASB^+^ cases and consider them contaminated samples. Assuming an inherent 10%–12% FN rate due to Gram-positive infections, combined with our protocol-specific FN rate of 5.6% with *E. coli* samples, we estimate the true FN rate of this protocol to be 15.6%–17.6%. Collectively, this technology, through a combination of color change, fluorescence, and turbidity, can detect up to 88%–90% of ASB-causing organisms (Fig. S8) (8, 13).

Urine dipstick is frequently used in low-resource settings to diagnose ASB, but it is generally an unreliable test (26). Similarly to previous studies (27), nitrite readings yielded a 68.8% FN rate, and leukocyte esterase resulted in a 43.8% FN rate in our obstetric clinical sample testing (Fig. 6). Our P/A kit protocol presented here demonstrates only a 5.6% FN rate in *E. coli in vitro* testing and a FN rate of 0% in 8/8 ASB^+^ obstetric clinical samples due to coliforms, which is much improved from current screening methods utilizing dipstick alone. The Aquagenx technology is not equipped to detect Gram-positive organisms. Our testing demonstrated this with clean-catch sample #1 (Fig. 4), which had a bacterial concentration of 10^6^ CFU/mL *Enterococcus* spp., and samples #15, #22, #27, #28, #30, and #37 from pregnant patients (Fig. 6), which had ≥10^5^ CFU/mL of Gram-positive species including *Staphylococcus* spp., *Streptococcus* spp., and *Corynebacterium* spp. The inclusion of these additional non-coliform samples brings the overall ASB^+^ FN rate to 50.0% (8/16 samples), still considerably better than current dipstick methods (3, 6). Though the leukocyte FN rate was slightly lower during clinical sample testing (43.8%), the FP rate was higher (54.5%). The development of complementary media to facilitate Gram-positive growth and turbidity is in our immediate future directions to reduce the FN rate of this protocol and enhance the medical applicability of this technology.

The P/A kit protocol involves the use of an inoculating loop transfer to a tube, requiring sterile technique and increasing the risk of human error. However, this is not unlike an at-home COVID-19 antigen test, which relies on self-collected swabs, suspension, and dilution onto a test strip (28). Although the MPN kit protocol eliminates the second dilution of the urine sample (D2), it still requires a second step (Step 2) in which the 100-mL sample is poured from one bag into another. We found this step to be more difficult to perform sterilely than D2 in the P/A kit protocol, and this contributed to the decision to proceed with P/A bags instead of MPN bags. In the future, a low-cost device could be developed to sterilely transfer accurate urine volumes for ASB diagnostic purposes to the Aquagenx bag for this protocol. Alternatively, a less sensitive version of the bag could be developed to require fewer manipulations of the urine sample prior to testing. In addition, the 24-hour waiting period could pose a challenge with contacting patients regarding results in low resource settings. While this is a significant barrier to treating maternal ASB, this issue is not isolated to Aquagenx diagnostics and must be more broadly addressed through public health intervention. Our work provides the foundation for the use of Aquagenx technology for medical diagnostic screening with limitless expansion opportunities, particularly in regions lacking microbial culture methods for species identification. The Aquagenx kit can be used with any sterile water source (bottled or boiled) and can be incubated in ambient temperature with no requirement for laboratory equipment (29). These qualities make this technology ideal for areas with limited resources. Aquagenx was, in fact, originally designed as a field test kit (26).

Future studies will aim to evaluate Aquagenx kits for the diagnosis of symptomatic UTI (>10^3^ CFU/mL) with a novel dilution protocol (30). Our MPN kit protocol could be useful for this purpose, as it already has shown great accuracy in enumerating bacterial loads > 10^3^ CFU/mL. Indeed, this added feature of MPN enumeration (17) may be more applicable to UTI than ASB, as determining the presence or absence of 10^5^ CFU/mL bacterial load is sufficient for ASB screening (1, 17). Aquagenx may also have utility in the diagnosis of other infections caused by *E. coli* and coliform species. We feel this technology and dilution protocol have the potential to expand access to ASB screening to millions of pregnant patients globally, especially those in disparate parts of the world. As of 2024, Aquagenx MPN kits cost between $7.80 and $10.00, while P/A kits can be purchased for as little as $5.70 USD per test (16). Indeed, the affordability and simplicity of the P/A kits are the main benefits over the MPN kits. Bulk purchases can further decrease the price point, making the kits even more cost-effective, especially in comparison to the average cost of $35 for urine culture. These low-cost kits could reduce global inequities in access to prenatal care and treatment, positively influencing disparate birth outcomes for mothers and infants (1, 7, 8). This is an affordable, feasible, and accurate test for developing nations and could have widespread future applications.

## MATERIALS AND METHODS

### Materials

Aquagenx P/A water quality test kits and Aquagenx MPN kits were used in all experiments with autoclave-sterilized deionized water (dH_2_O) or sterile human urine as the medium. Aquagenx uses a proprietary growth medium that functions by turning the sample blue/green when *E. coli* is present and metabolizes a glucose substrate. It fluoresces when coliforms metabolize a fluorogenic galactosidase substrate, indicating their presence (16).

Inoculating loops tested were Globe Scientific rigid sterile individually wrapped 1-µL/10 µL combination loops and Argos Technologies polystyrene sterile individually wrapped disposable 10-µL loops.

### Bacterial strains, media, and culture conditions

*E. coli* CFT073 was isolated from the blood and urine of a hospitalized patient with acute pyelonephritis (15) and was used for all studies unless otherwise noted. See Table 1 for a complete list of all bacterial species and strains used. Cultures were grown overnight in LB, which contains the following per liter: 0.5 g NaCl, 5 g yeast extract, and 10 g tryptone. Single colonies were inoculated and grown at 37°C under aerated conditions. Fifteen grams per liter of agar was added for LB agar plates.

**TABLE 1:** Bacterial strains used in this study

Genus and species	Strain	Reference
*E. coli*	CFT073	(31)
*E. coli*	ABU83972	(32)
*E. coli*	UTI89	(33)
*E. coli*	EFC2	(34)
*Klebsiella pneumoniae*	KPPR1	(35)
*Serratia marcescens*	UMH9	(36)
*Citrobacter freundii*	UMH14	(37)
*Acinetobacter baumannii*	AB0057	(38)
*P. mirabilis*	HI4320	(39)
*Pseudomonas aeruginosa*	PAO1	ATCC 15692
Carbapenem-resistant *Enterobacter cloacae*	CRE14	unpublished

Bacterial inputs for mock samples were generated as follows: overnight culture densities were determined at OD_600_ nm for conversion to concentrations in CFU/mL. Bacterial concentrations were normalized to targeted inputs (10^3^, 10^4^, and 10^5^ CFU/mL) in 50 mL of sterile solvent (water or urine). This sample was plated to enumerate actual CFU/mL concentration and used for downstream testing.

### General Aquagenx kit protocol

Aquagenx MPN kit instructions (17) were followed. In a Thio-Bag, 100 mL of sterile water, loop inoculation, and growth medium were added and shaken (Step 1). The full contents of the Thio-Bag were then poured into a Compartment Bag (Step 2), distributing the liquid as evenly as possible into all five compartments, and the bag was sealed and incubated for 20 hours at 37°C.

The P/A test kit instructions for use provided by Aquagenx (22) were followed. In a P/A bag, 100 mL of sterile water, loop inoculation, and Aquagenx growth medium were added. The contents of the bag were shaken to mix; then, the bag was sealed and incubated for 20 hours at 37°C.

Both MPN and P/A kits were scored in ambient and UV light, where a positive result was determined by blue/green color in ambient light, fluorescence under UV light, or a change in the media from transparent to opaque (turbidity).

### Human urine samples

All clean-catch collection human urine was from de-identified donors of the female sex who were <35 years of age, not menstruating, had not taken antibiotics in the last 2 weeks, and were not experiencing UTI symptoms at the time of donation. The urine cups were refrigerated quickly and tested within 24 hours of donation. Unfiltered human urine was tested for interference with Aquagenx growth medium. Sterile urine used was filter sterilized (0.22 µM) after pooling together >6 clean-catch donations.

De-identified urine samples were collected from the University of South Alabama Hospital. First, UTI^+^ (*n* = 5) and UTI^−^ (*n* = 5) urine samples from females were chosen and screened for ASB^+^ bacterial load using the Aquagenx protocol. These samples were preserved in boric acid in gray top collection tubes, refrigerated, and tested within 48 hours of collection. Urine samples sent for culture from the University of South Alabama OB/GYN department were screened for positive pregnancy status. Samples from pregnant individuals (*n* = 40) were collected from urine cups, refrigerated, and screened for ASB within 48 hours of collection.

Urobiome testing was performed by spread and drip plating clinical samples to enumerate bacterial load (CFU/mL). UTI^+^ and UTI^−^ samples were plated on LB agar, and samples from pregnant patients were plated on both LB agar and 5% sheep blood agar plates. Identification of each species was determined via mass spectrometry (MALDI-TOF) provided by the University Hospital.

### MPN kit protocol optimization

To optimize the urine dilution in Step 1, three inoculating loop volumes were tested for accuracy in transferring 1 CFU from 10^3^ CFU/mL samples into the Thio-Bag. Each time, an inoculating loop was transferred from the input sample onto an LB agar plate and struck out for enumeration. Next, the protocol was validated using mock urine. Inputs were plated on LB agar to determine the actual inoculum concentrations (CFU/mL). These inputs were subjected to the MPN bag protocol, where a 1-µL inoculating loop was transferred from the sample to the Thio-Bag in Step 1. The Aquagenx MPN color chart (17) was then used to determine the MPN/100 mL value for each sample.

### P/A kit protocol optimization

A simple urine dilution protocol was developed for P/A kit ASB screening using sterile plastic inoculating loops and a 2-mL microtube. To optimize the protocol, various combination inoculating loop volumes were tested in D1 (Fig. S4) and D2 (Fig. S5) via spread plating and streak plating onto LB agar plates. Relative accuracies of different loop volumes (10 µL, 10 µL × 2) in D1 were determined by comparing CFU counts from spread plating 100 µL of the diluted sample in the microtubes. The target D1 plate counts for ASB^+^ and ASB^−^ urine samples were 100 CFU and 10 CFU, corresponding to the goal microtube concentrations of 10^3^ and 10^2^ CFU/mL, respectively. D2 was optimized by streaking out various loop volumes (1 µL, 10 µL, 10 µL × 2) from the microtube onto LB agar plates, simulating the P/A bag inoculation process with a goal of 1–10 CFU plate count for ASB^+^ urine and 0 CFU for ASB^−^ urine.

### Statistics

FP and FN rates were calculated as follows: FPR = FP/(FP + TN) and FNR = FN/(FN + TP). Sensitivity, specificity, positive predictive value, and negative predictive values were determined using GraphPad Prism v10. Sensitivity and specificity were determined via Wilson/Brown analysis. Fishers exact test was used to compute 95% CI for Prism statistical values. To determine patient sample sizes, 10 patient samples (Fig. 5) were initially tested for preliminary data. These values were used to compute the overall clinical sample size needed via power analysis at 80% power with an alpha of 0.05, which was determined to be *n* = 21. A retrospective analysis was then performed at a higher power (90%) after testing 30 obstetric samples (Fig. 6) and determined the sample size to be *n* = 33.

## SUPPLEMENTAL MATERIAL

Data Set S1 aem.01567-24-s0001.xlsx De-identified patient sample data.

Supplemental figures aem.01567-24-s0002.pdf Figures S1 to S8.

ASM does not own the copyrights to Supplemental Material that may be linked to, or accessed through, an article. The authors have granted ASM a non-exclusive, world-wide license to publish the Supplemental Material files. Please contact the corresponding author directly for reuse.
