# Supplementary material for: Asymptomatic bacteriuria screening for developing countries using a modified water quality test kit
Source: Appl Environ Microbiol. 2024 Oct 30;90(11):e01567-24. doi: 10.1128/aem.01567-24 (PMC11577777; doi:10.1128/aem.01567-24)
Supplement: Supplemental figures — Figures S1 to S8. [file aem.01567-24-s0002.pdf]

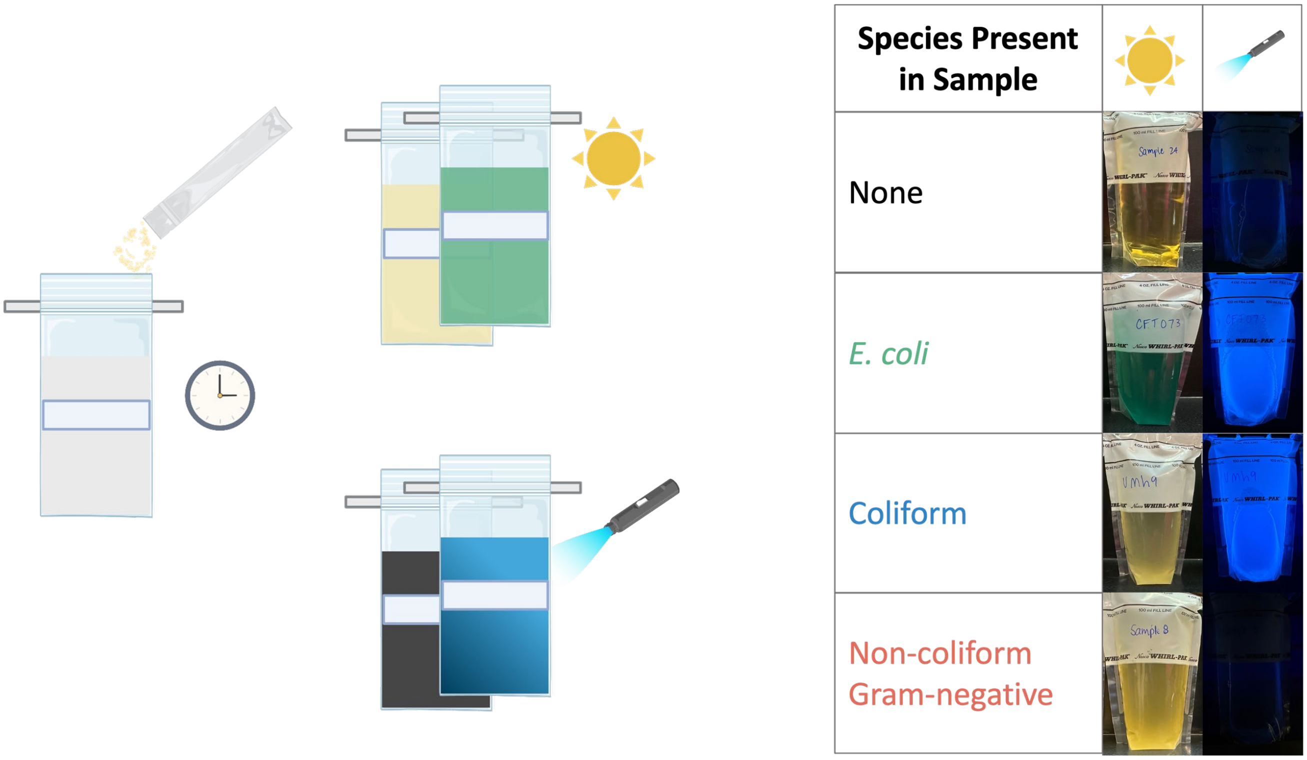

Supplemental Figure 1. Detection of ASB causative species with Aquagenx® technology. Aquagenx® test results in ambient and UV light for negative, *E. coli*⁺, coliform⁺, and non-coliform Gram-negative⁺ urine samples [1].

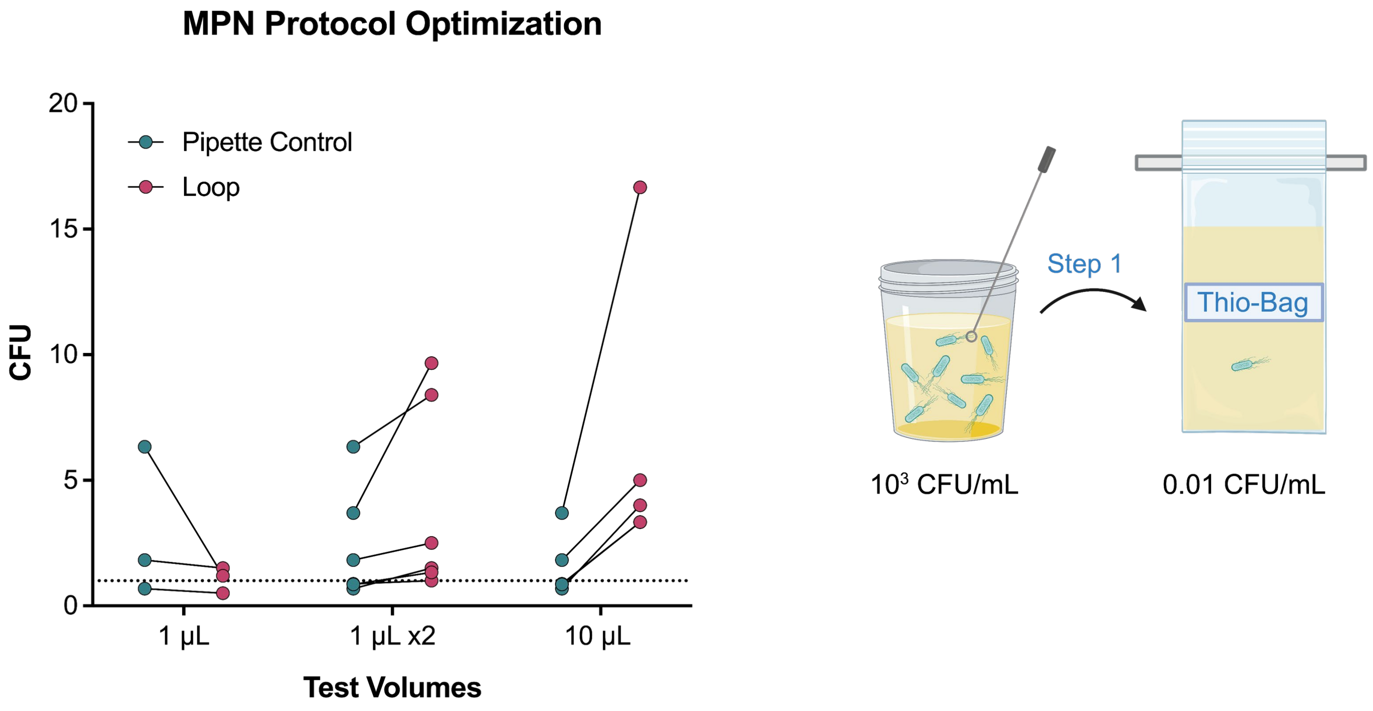

Supplemental Figure 2. Testing dilution from mock urine into thio bags for MPN kit protocol. In order to achieve a single dilution ASB screening protocol using MPN kits, we proposed the transfer of an inoculating loop to get 10^3 CFU/mL urine at the lower detectable range of the bag (1 CFU). To optimize this protocol at the lower limit of detection, mock ~10^3 CFU/mL *E. coli* urine samples were prepared and tested. From each sample, multiple inoculating loop volumes, 1 µL, 1 µL x2, or 10 µL, were struck out on LB agar plates to determine which volume best achieves a transfer of 1 CFU into the Thio-Bag (dashed black line). From each mock urine, a pipette volume of 1 µL was platted as a control. Inoculating loop CFU values (pink circles) are attached to their corresponding input (green circles).

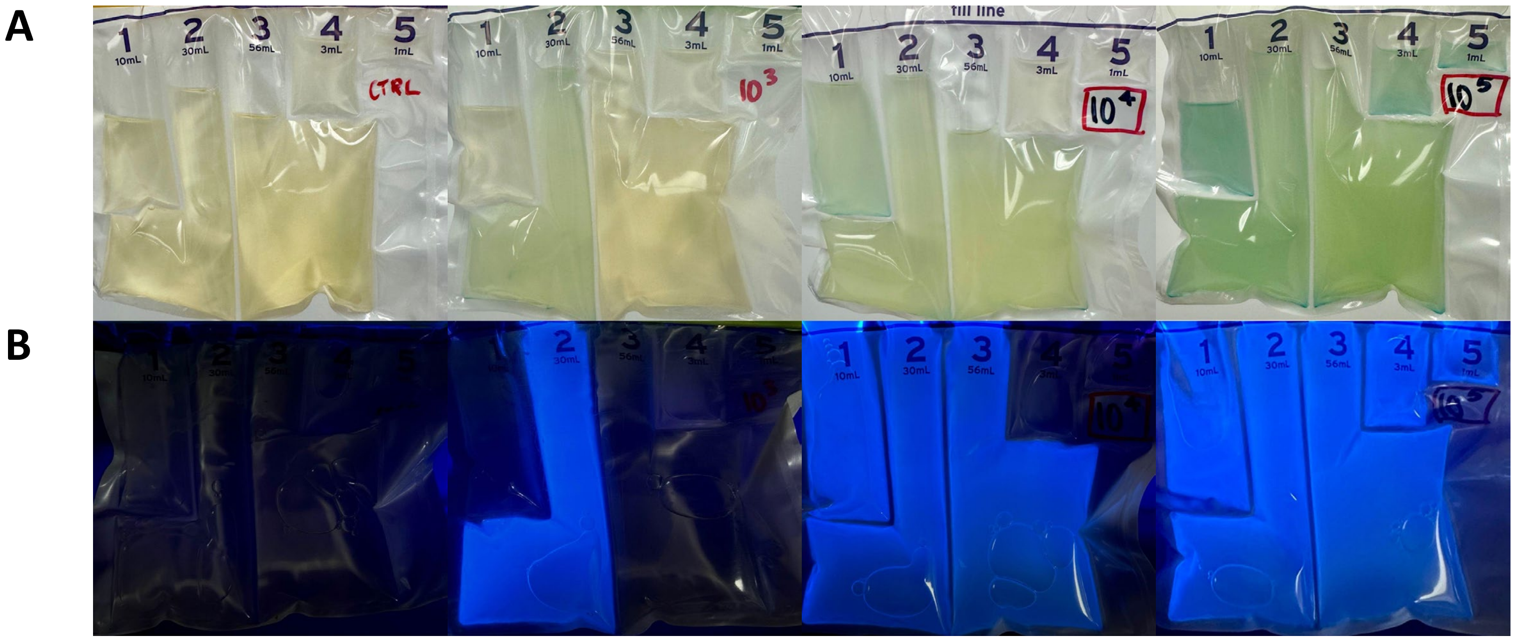

Supplemental Figure 3. Images of Aquagenx® MPN bag ASB screening results.
Mock *E. coli* urine samples were prepared at clinically relevant concentrations and subjected to our MPN bag ASB screening protocol [2]. The bags were observed in **(A)** ambient and **(B)** UV light.

Row Number	Compartment Number					MPN/ 100 mL	Test Sample CFU/mL
	1	2	3	4	5		
1						0.0	< 1.00 x 10^3
2						1.0	1.00 x 10^3
3						1.0	1.00 x 10^3
4						1.1	1.10 x 10^3
5						1.2	1.20 x 10^3
6						1.5	1.50 x 10^3
7						2.0	2.00 x 10^3
8						2.1	2.10 x 10^3
9						2.1	2.10 x 10^3
10						2.4	2.40 x 10^3
11						2.4	2.40 x 10^3
12						2.6	2.60 x 10^3
13						3.2	3.20 x 10^3
14						3.7	3.70 x 10^3
15						3.1	3.10 x 10^3
16						3.2	3.20 x 10^3
17						3.4	3.40 x 10^3
18						3.9	3.90 x 10^3
19						4.0	4.00 x 10^3
20						4.7	4.70 x 10^3
21						5.2	5.20 x 10^3
22						5.4	5.40 x 10^3
23						5.6	5.60 x 10^3
24						5.8	5.80 x 10^3
25						8.4	8.40 x 10^3
26						9.1	9.10 x 10^3
27						9.6	9.60 x 10^3
28						13.6	1.36 x 10^4
29						17.1	1.71 x 10^4
30						32.6	3.26 x 10^4
31						48.3	4.83 x 10^4
32						>100	> 1.00 x 10^5

Supplemental Figure 4. Interpreting screening results with Aquagenx® MPN kits.
Aquagenx® color chart [2] MPN/100mL values were converted to expected bacterial load of the test sample assuming a 1:100,000 sample dilution via our protocol.

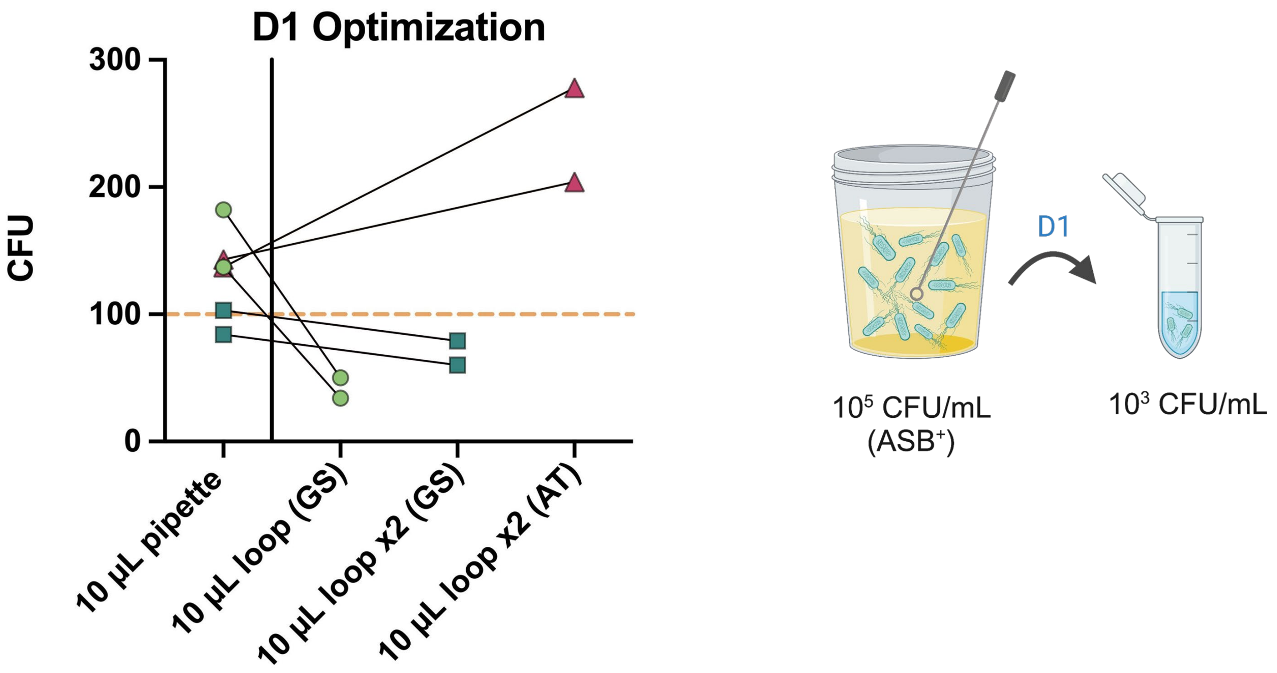

Supplemental Figure 5. Testing P/A kit protocol D1 dilution with mock ASB urine samples. In order to get a clinical ASB⁺ sample (10^5 CFU/mL) within the detectable range of the Aquagenx® P/A kit [3], we tested a series of dilution protocols. The first dilution step (D1) from the urine specimen to the microtube was optimized by testing multiple inoculating loop combinations from 10^5 CFU/mL (ASB^+) urine to the microtube. A Globe Scientific® (GS) 10 µL loop was tested with one transfer to the microtube (green circles, n=2) and with two transfers (blue squares, n=2). A second brand of 10 µL loop, Argos Technologies™ (AT), was also tested with two transfers (red triangles, n=2). Each time, a 10 µL volume was pipetted from the same urine sample as an internal control. CFU were determined by spread plating 100 µL from the microtube onto LB agar plates. The target plate colony count was 100 CFU (dashed orange line), representative of a positive ASB screening result.

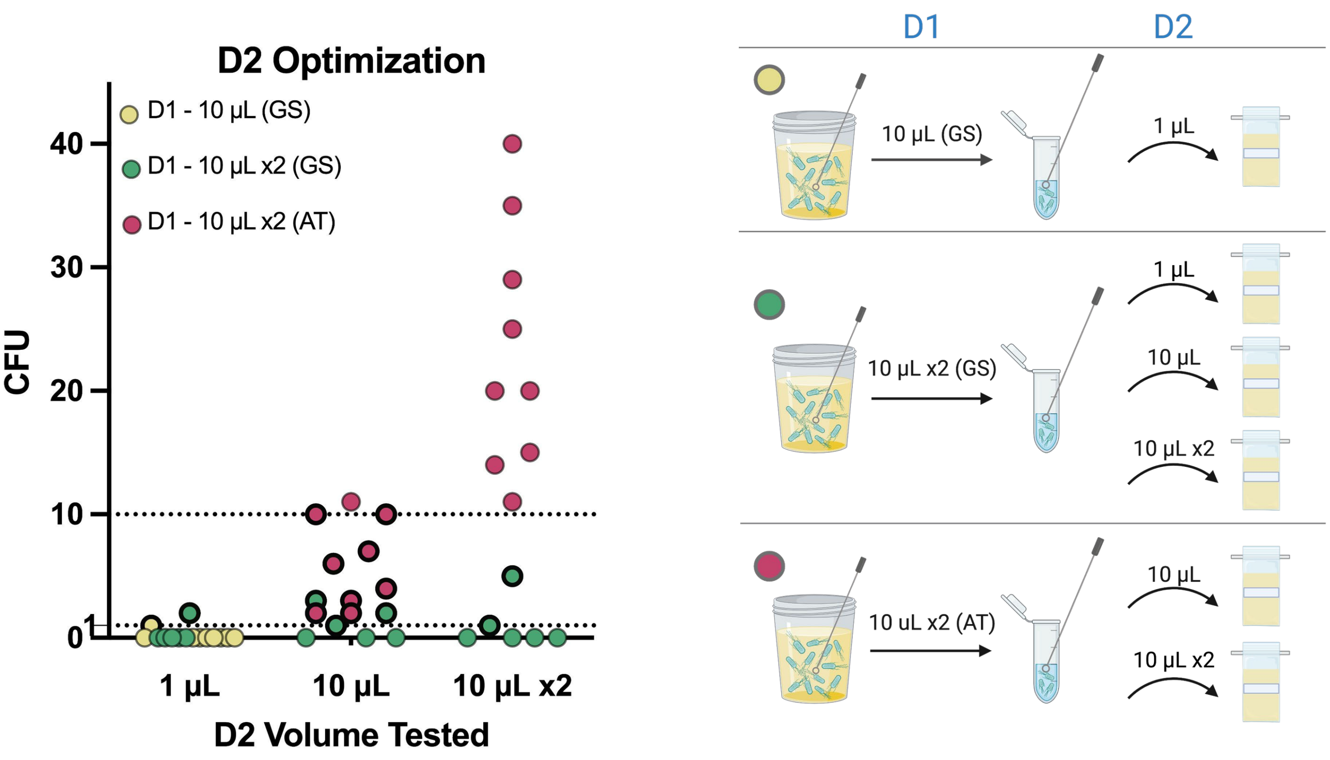

Supplemental Figure 6. Testing P/A kit D2 dilution from mock *E. coli* ASB+ urine samples. Multiple dilution 1 (D1) and dilution 2 (D2) inoculating loop combinations were tested with mock ASB+ (10^5 CFU/mL) urine samples to determine which protocol yielded the lowest false negative rate while remaining as close to the lower limit of the bag (1 CFU) as possible. Three different D1 volumes were tested in dilution from urine sample to microtube: Globe Scientific© (GS) 10 µL (yellow), GS 10 µL x 2 (green), and Argos Technologies™ (AT) 10 µL x 2 (pink). From the microtubes, we struck out 1 µL, 10 µL, and 10 µL twice with inoculating loops on LB agar plates to simulate D2 in Aquagenx® bags. Our target plate count was 1-10 CFU, indicated by the dashed lines. Bolded symbols indicate values within the desired CFU range.

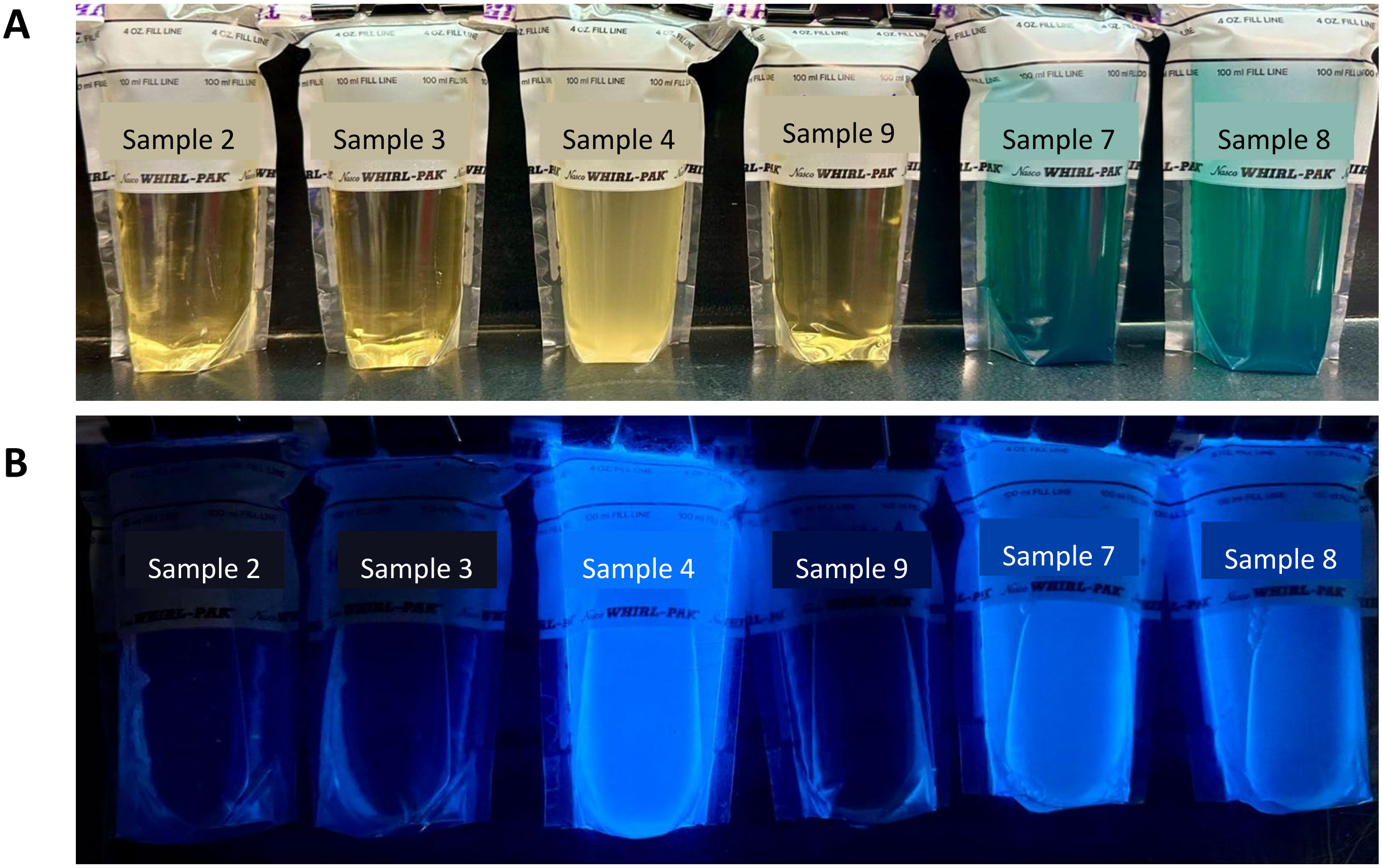

Supplemental Figure 7. Images of Aquagenx® P/A kit ASB screening results.
Aquagenx® P/A kits [3] were used on clinical samples (Fig. 5) and observed in **(A)** ambient and **(B)** UV light.

Causative species	Percentage of ASB Cases	Color Change	Fluorescence	Turbidity
E. coli	61.51%	✓	✓	✓
Klebsiella spp.	17.03%	X	✓	✓
Pseudomonas spp.	7.57%	X	X	✓
Proteus spp.	3.78%	X	X	✓
Enterococcus spp.	5.36%	X	X	X
Staphylococcus spp.	4.73%	X	X	X

Supplemental Figure 8. Detection of top ASB causative species with Aquagenx® technology. Top ASB causative species [4] were tested with Aquagenx® P/A kits and scored by colorimetric change, fluorescence under UV light, and turbidity of the media. Aquagenx® technology detected 3/5 species that collectively account for 90.05% of ASB cases.

References

1. Aquagenx®. *Aquagenx.com*. [cited 2024; Available from: https://www.aquagenx.com/buy-from-aquagenx/.
2. Aquagenx®, *Most Probable Number (MPN) Kit Instructions for Use: Drinking Water*. 2013.
3. Aquagenx®, *Presence/Absence (P/A) Kit Instructions for Use: Drinking Water*. https://www.aquagenx.com/product-documentation/.
4. S. Khan, R., P. Singh, Z. Siddiqui, M. Ansari, *Pregnancy-associated asymptomatic bacteriuria and drug resistance.* Journal of Taibah University Medical Sciences, 2015. **10**(3): p. 340-345.
